# Implication of platelets and complement C3 as link between innate immunity and tubulointerstitial injury in renal vasculitis with MPO-ANCA seropositivity

**DOI:** 10.3389/fimmu.2022.1054457

**Published:** 2022-11-11

**Authors:** Eva Baier, Désirée Tampe, Ingmar Alexander Kluge, Samy Hakroush, Björn Tampe

**Affiliations:** ^1^ Department of Nephrology and Rheumatology, University Medical Center Göttingen, Göttingen, Germany; ^2^ Institute of Pathology, University Medical Center Göttingen, Göttingen, Germany; ^3^ SYNLAB Pathology Hannover, SYNLAB Holding Germany, Augsburg, Germany

**Keywords:** innate immunity, platelets, complement system, complement C3, ANCA-associated renal vasculitis, MPO-ANCA, PR3-ANCA, tubulointerstitial injury

## Abstract

**Introduction:**

Antineutrophil cytoplasmic antibody (ANCA)-associated vasculitis (AAV) is a potentially life-threatening systemic small-vessel vasculitis that is characterized by pauci-immune glomerulonephritis, depicting in turn a major denominator of AAV mortality. It is well established that AAV patients feature an increased risk of developing thrombotic events, and platelets are activated in AAV patients being triggered by the alternative complement pathway. Platelets guard vessels integrity and initiate thrombus formation in response to endothelial damage, further constituting a triangular interconnection with the activation of neutrophils and the complement system. We here aimed to systematically assess the relevance of platelet counts and systemic complement system activation regarding distinct histopathological lesions in ANCA-associated renal vasculitis.

**Methods:**

A cohort of 53 biopsy-proven cases of ANCA-associated renal vasculitis were retrospectively enrolled in a single-center observational study. Univariate and multivariate regression analysis was performed to identify parameters associated with platelet counts in ANCA-associated renal vasculitis compared to disease controls. Finally, the relevance of platelets for disease course and recovery was assessed by survival analysis.

**Results:**

Lower platelet counts correlated with markers of kidney injury including eGFR loss (*p=0.0004*) and lower complement C3 levels (*p=0.0037*). Multivariate and subgroup analysis revealed that this association was only present in the subgroup with MPO-ANCA seropositivity (eGFR loss: *p=0.0009*, lower C3: *p=0.0032*). While lower platelet counts correlated with kidney injury in the PR3-ANCA subgroup (eGFR loss: *p=0.0272*), we did not observe an independent association with complement C3 levels (*p=0.4497*). Independent of any glomerular lesion, lower platelet counts correlated with interstitial fibrosis (*p=0.0313*), tubular atrophy (*p=0.0073*), and tubulitis in areas of interstitial fibrosis and tubular atrophy (*p=0.0033*). Finally, we observed significant differences with increased requirement of kidney replacement therapy (KRT) or death in the subgroup below median platelet counts (HR: 4.1, 95% CI: 1.6-10, *p=0.0047*), associated with a lower probability of discharge and prolonged hospitalization in this subgroup (HR: 0.5, 95% CI: 0.3-0.9, *p=0.0113*).

**Conclusion:**

Based on our observation that an association between platelets and complement system activation is only observed in the MPO-ANCA subgroup, this could implicate that platelets and complement C3 link innate immunity to tubulointerstitial injury in the presence of MPO-ANCA autoantibodies.

## Introduction

Antineutrophil cytoplasmic antibody (ANCA)-associated vasculitis (AAV) is a potentially life-threatening systemic small-vessel vasculitis that is characterized by pauci-immune glomerulonephritis in case of kidney involvement, depicting in turn a major denominator of AAV mortality (1–4). Two principal antigens on neutrophils, namely proteinase 3 (PR3) and myeloperoxidase (MPO), provide epitopes for ANCA binding, thus promoting neutrophil activation and neutrophil extracellular traps (NETs) formation (“NETosis”), which consists of the extrusion of lattice-like chromatin fibers harboring cytokines and antimicrobial proteins that contribute to host defense under physiological conditions and promote endothelial damage and vascular inflammation culminating in necrotizing vasculitis in the context of autoimmunity (5–7). ANCA-stimulated neutrophils have been shown to induce the formation of NETs, which contain the ANCA target antigens MPO and PR3 (8). In ANCA-associated renal vasculitis, NETs are located in close proximity to neutrophil infiltrates in affected glomeruli and tubulointerstitium (8). NETs are capable to activate the classical complement pathway due to the interaction with C1q (9). Neutrophils itself contain various components of the alternative but not the classical complement pathway (10). Particularly in AAV with activated neutrophils by ANCA autoantibodies, the alternative dominates over the classical complement pathway (11). Therefore, the involvement of the alternative pathway of the complement system captures a pathophysiological key role in NETosis and more generally in AAV, which is impressively constrained by the efficacy of avacopan, a novel drug targeting the C5a receptor (12–14). As part of innate immunity, the complement system is composed of approximately more than thirty serum proteins, whose pathways are either classical-, alternative- or lectin-categorized (15, 16). Depending on different induction modes, a self-reinforcing domino-effect-like cascade is initiated that disembarks in the common formation of the so-called membrane attack complex (MAC), wherein also inhibitory regulatory mechanisms are interposed, such as factor H suppressing the activation of the alternative pathway by preventing C3b opsonization, which in turn was shown to affect NETosis (17, 18).

Being influenced by both components, complement system activation and neutrophils/NETosis, the coagulation system is another relevant pathophysiological factor acting on the injured endothelium as the primary side of inflammation (18, 19). It is well established that AAV patients feature a two- to three-fold increased risk of developing thrombotic events, and platelets are activated in AAV patients being triggered by the alternative complement pathway (20–26). Platelets guard vessels integrity and initiate thrombus formation in response to endothelial damage, further constituting a triangular interconnection with the activation of neutrophils, formation of NETs and the complement system (26–28). Although ANCA autoantibody activity is an established inducer of NETs, its presence is not always consistent with disease activity (29). Regarding additional mechanisms of neutrophil activation, recent reports suggested that platelets itself can also regulate formation of NETs in AAV (30). However, the implication of platelets regarding vasculitis manifestation and complement system activation in ANCA-associated renal vasculitis has not been described yet. Therefore, we here aimed to systematically assess the relevance of platelet counts and systemic complement system activation regarding distinct histopathological lesions in ANCA-associated renal vasculitis.

## Methods

### Study population and subgroup formation

A well characterized cohort of 53 biopsy-proven cases of ANCA-associated renal vasculitis were retrospectively enrolled between 2015 till 2020 in a single-center observational study at the University Medical Center Göttingen, Göttingen, Germany (**Supplementary Table 1**) (31–35). In addition, 18 cases with IgA nephropathy, 20 with diabetic kidney disease, and 27 with acute interstitial nephritis were included as disease controls (**Supplementary Table 2**). While no formal approval was required for the use of routine clinical data, a favorable ethical opinion was granted by the local Ethics committee (no. 22/2/14 and 28/09/17). All participants provided their written informed consent for the utilization of routinely collected data for research purposes as part of their regular medical care. Medical records were used to collect data on age, sex, medication, comorbidities, laboratory findings at admission (creatinine, estimated glomerular filtration rate/eGFR, blood urea nitrogen/BUN, potassium, albumin, aspartate-amino transferase/AST, alkaline phosphatase/AP, gamma glutamyl transferase/γGT, bilirubin, complement C3 and C4), at the time of kidney biopsy (platelets, hemoglobin, white blood cells/WBC, C-reactive protein/CRP), dates of admission and discharge from hospital. The Birmingham Vasculitis Activity Score (BVAS) was assessed as previously described (36).

### Renal histopathology

Renal pathologists evaluated all kidney biopsies and was blinded to clinical data analysis. Based on the current version of the Banff scoring system for renal allograft pathology, tubulointerstitial lesions were scored as previously reported: arteriolar hyalinosis (*ah*), arteritis (*v*), glomerulitis (*g*), inflammation in areas of interstitial fibrosis and tubular atrophy (*i-IFTA*), interstitial fibrosis (*ci*), interstitial inflammation (*i*), peritubular capillaritis (*ptc*), total inflammation (*ti*), tubular atrophy (*ct*), tubulitis (*t*), and tubulitis in areas of interstitial fibrosis and tubular atrophy (*t-IFTA*) (37, 38). Tubular injury lesions were systematically assessed as recently described (39). Briefly, tubular dilation, tubular necrosis, epithelial simplification, non-isometric cell vacuolization, red blood cell (RBC) and necrotic casts were scored with a range from 0 to 4 depending on the fraction of affected cortical area of renal biopsy (score 0: <1%, 1: ≥1-10%, 2: ≥10-25%, 3: ≥25-50%, 4: >50%). Moreover, all injured glomeruli (crescentic or/and necrotic) were screened for the presence of a Bowman’s capsule rupture, whose extent was further quantified as previously described (40–42).

### Remission induction therapy

Steroids were administered either as intravenous pulse therapy or orally with a tapering schedule. At time of platelet measurement and kidney biopsy, all patients received steroids and further remission induction therapy was initiated thereafter based on histopathological confirmation of ANCA GN. Plasma exchange (PEX) was administered during the induction period at the discretion of treating physicians. Rituximab (RTX) was administered as four intravenous doses at 375 mg/m^2^ every week; RTX was not administered within 48 hours before PEX treatment. Cyclophosphamide (CYC) was administered as three intravenous doses up to 15 mg/kg every 2 weeks and every 3 weeks thereafter, adjusted for age and renal function. Combination therapy was administered as four intravenous doses at 375 mg/m^2^ RTX every week and two intravenous doses at 15 mg/kg CYC every 2 weeks. On the discretion of treating physicians, choice of remission induction therapy was dependent on previous regimens and individual patients, more likely to choose RTX in younger patients with toxicity being the main reason for this choice (**Supplementary Table 1**) (43). Prophylaxis to prevent pneumocystis (carinii) jiroveci infection was administered according to local practice.

### Statistical analysis

Normally distributed values are presented as mean ± standard deviation (SD), while non-normally distributed parameters are shown as median and interquartile range (IQR). Normal distribution was evaluated by Shapiro-Wilk testing. Categorical variables as percentages of total. Statistical comparisons were not formally powered or prespecified. Probability values (*p* value) below 0.05 were considered statistically significant. For normally distributed values, mean comparisons were performed with unpaired student’s t-test, while estimation plots were used for data visualization. For non-normally distributed values, median comparisons were performed with the Mann-Whitney-U-test. Heatmaps reflect the mean values of Spearman’s ρ in the univariate linear regression analysis, circle size represents significance level. Survival-curve analyses were performed using the Kaplan-Meier method, wherein log rank (Mantel-Cox) testing was conducted for curve comparison. Time-to-event was registered and discharge from hospital was defined as event. Results are shown as hazard ratio (HR) and 95% confidence interval (CI). For stepwise multiple linear regression, covariates were retained to significant differences in the linear regression model to avoid model over-fit. Data analyses were performed with GraphPad Prism (version 9.4 for MacOS, GraphPad Software, San Diego, California, USA) and IBM SPSS Statistics (version 28 for MacOS, IBM Corporation, Armonk, NY, USA).

## Results

### Lower platelet counts associate with kidney injury and lower complement C3 levels in MPO-ANCA-associated renal vasculitis

**Figure 1:**
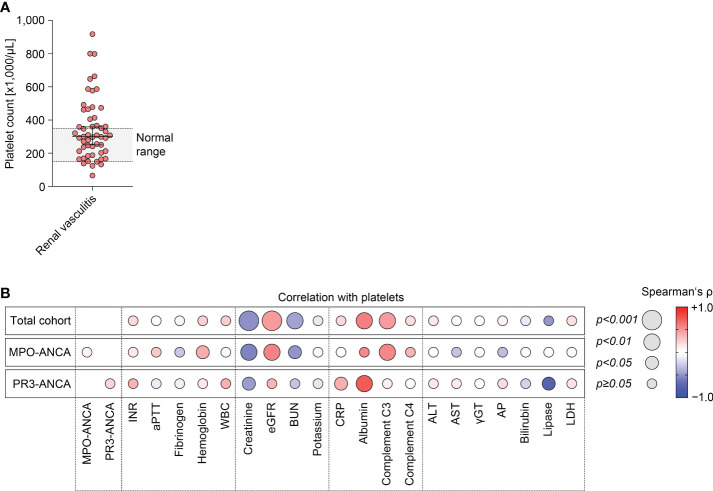
Lower platelet counts associate with kidney injury and lower complement C3 levels in ANCA-associated renal vasculitis. **(A)** Distribution of platelet counts in the total cohort of ANCA-associated renal vasculitis (normal range: 150,000-350,000/µL). **(B)** Correlations between platelet counts and laboratory parameters in ANCA-associated renal vasculitis are shown by heatmap reflecting mean values of Spearman’s ρ, circle size represents significance level. ALT, alanine aminotransferase; ANCA, antineutrophil cytoplasmic antibody; AP, alkaline phosphatase; aPTT, activated partial thromboplastin time; AST, aspartate amino transferase; BUN, blood urea nitrogen; CRP, C-reactive protein; eGFR, estimated glomerular filtration rate (CKD-EPI); INR, international normalized ratio; LDH, lactate dehydrogenase; MPO, myeloperoxidase; PR3, proteinase 3; WBC, white blood cells; γGT, gamma glutamyl transferase.

The study conduction is summarized in **Supplementary Figure 1**, the baseline characteristics of the total cohort are shown in **Supplementary Table 1**. Median (IQR) platelet counts were 300,000/µL (207,000-438,000/µL), therefore most patients were within or above the normal range (150,000-350,000/µL, **Figure 1A**). Univariate analysis revealed that lower platelet counts correlated with markers of kidney injury, including serum creatinine levels (ρ=-0.51, *p<0.0001*), eGFR loss (ρ=0.47, *p=0.0004*), and BUN (ρ=-0.44, *p=0.0025*, **Figure 1B**). Furthermore, lower platelet counts were also associated with reduced levels of serum albumin (ρ=0.59, *p=0.0020*) and complement C3 (ρ=0.46, *p=0.0037*, **Figure 1B**). By contrast, we observed no association between platelet counts and ANCA autoantibody levels, coagulation parameters, other markers of systemic inflammation, or parameters indicative for liver injury (**Figure 1B**). Multivariate analysis confirmed an independent association between platelet counts, kidney injury (eGFR loss: *p=0.0005*) and lower levels of complement C3 (*p=0.0416*) in the total cohort of ANCA-associated renal vasculitis (**Table 1**). Moreover, BUN itself was not the main denominator associated with lower platelet counts (*p=0.4011*, **Table 1**), implicating distinct mechanisms independent of uremia in ANCA-associated renal vasculitis. This association was equally detectable in the subgroup with MPO-ANCA seropositivity (eGFR loss: *p=0.0009*, lower complement C3: *p=0.0032*, **Table 1**). While lower platelet counts correlated with kidney injury in renal vasculitis with PR3-ANCA seropositivity (eGFR loss: *p=0.0272*), we did not observe an independent association with complement C3 levels in this subgroup (*p=0.4497*, **Table 1**). To validate that these findings are specific for ANCA-associated renal vasculitis, we next analyzed platelet counts, markers of kidney injury and complement levels in disease controls including IgA nephropathy, diabetic kidney disease, and acute interstitial nephritis (**Supplementary Table 2**). We did not observe any significant association between platelet counts and serum creatinine levels (ρ=-0.23, *p>0.05*), eGFR (ρ=0.24, *p>0.05*), complement C3 (ρ=0.07, *p>0.05*) or C4 (ρ=0.06, *p>0.05*, **Supplementary Figure 2**). In summary, lower platelet counts associated with kidney injury in ANCA-associated renal vasculitis. Furthermore, lower platelet counts were independently correlated with lower complement C3 levels specifically in patients with MPO-ANCA seropositivity that was not confirmed in the PR3-ANCA subgroup. Finally, there was no association with unspecific markers of systemic inflammation in ANCA-associated renal vasculitis.

**Table 1:** Stepwise multiple linear regression analyses with platelet counts as the dependent variable.

Total cohort	β	p value
Creatinine – mg/dL eGFR – mL/min/1.73 m^2^ BUN – mg/dL Albumin – g/dL Complement C3 – g/L	-0.0107 0.6405 -0.1567 0.1056 0.3276	*0.9645* *0.0005* *0.4011* *0.5109* *0.0416*
*MPO-ANCA*		
eGFR – mL/min/1.73 m^2^ Albumin – g/dL Complement C3 – g/L	0.6784 -0.0597 0.5375	*0.0009* *0.7791* *0.0032*
*PR3-ANCA*		
eGFR – mL/min/1.73 m^2^ Albumin – g/dL Complement C3 – g/L	0.7247 0.2595 0.2443	*0.0272* *0.4013* *0.4497*

ANCA, antineutrophil cytoplasmic antibody; BUN, blood urea nitrogen; eGFR, estimated glomerular filtration rate (CKD-EPI); MPO, myeloperoxidase; PR3, proteinase 3.

### Lower platelet counts associate specifically with tubulointerstitial injury in ANCA-associated renal vasculitis

**Figure 2:**
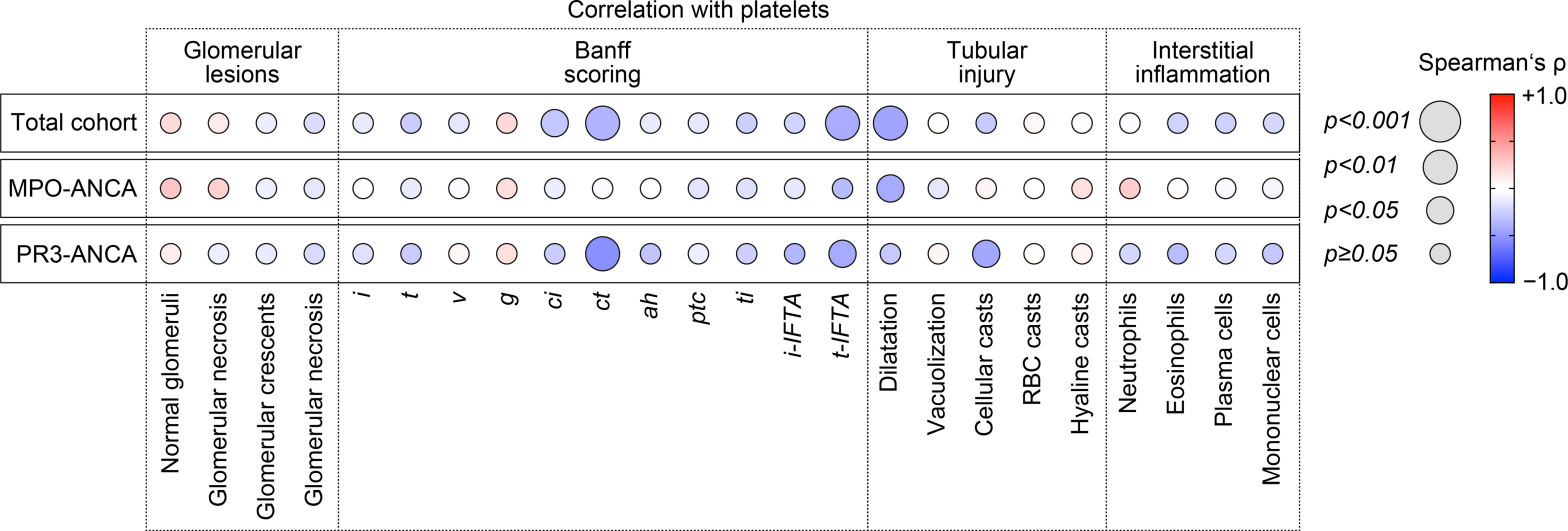
Lower platelet counts associate specifically with tubulointerstitial injury in ANCA-associated renal vasculitis. Correlations between platelet counts and histopathological lesions in ANCA-associated renal vasculitis are shown by heatmap reflecting mean values of Spearman’s ρ, circle size represents significance level. *ah*, arteriolar hyalinosis; ANCA, antineutrophil cytoplasmic antibody; *ci*, interstitial fibrosis; *ct*, tubular atrophy; *g*, glomerulitis; *i*, interstitial inflammation; *i-IFTA*, inflammation in areas of interstitial fibrosis and tubular atrophy; MPO, myeloperoxidase; PR3, proteinase 3; *ptc*, peritubular capillaritis; RBC, red blood cell; *t*, tubulitis; *ti*, total inflammation; *t-IFTA*, tubulitis in areas of interstitial fibrosis and tubular atrophy; *v*, intimal arteritis.

Because we have observed an association between lower platelet counts and laboratory markers of kidney injury, we next analyzed the association between platelet counts and histopathological lesions in ANCA-associated renal vasculitis. Interestingly, there was no association between platelet counts and any glomerular lesion in ANCA-associated renal vasculitis (**Figure 2**). By contrast, lower platelet counts correlated with interstitial fibrosis (*ci*, *p=0.0313*), tubular atrophy (*ct*, *p=0.0073*), and tubulitis in areas of interstitial fibrosis and tubular atrophy (*t-IFTA*, *p=0.0033*, **Figure 2**). Detailed morphological analysis of tubular injury indicated that lower platelet counts correlated specifically with tubular dilatation in renal vasculitis with MPO-ANCA seropositivity (*p=0.0347*), while an association with cellular casts was observed in the PR3-ANCA subgroup (*p=0.0352*, **Figure 2**). By contrast, we did not observe any association with inflammatory lesions including intrarenal immune cell infiltration (**Figure 2**). In summary, lower platelet counts correlated specifically with tubulointerstitial injury in ANCA-associated renal vasculitis. Moreover, we again observed differences between ANCA subtypes regarding distinct tubular injury patterns.

### Disease course and recovery in ANCA-associated renal vasculitis differs according to platelet counts

**Figure 3:**
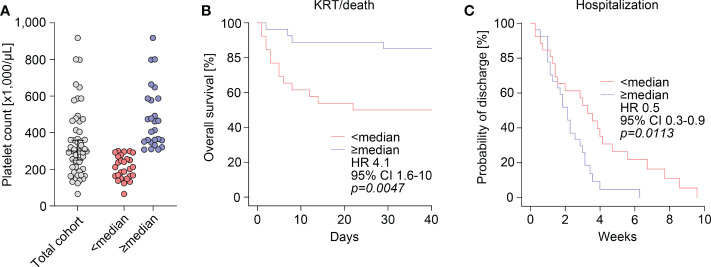
Disease course and recovery in ANCA-associated renal vasculitis differs according to platelet counts. **(A)** The scatter dot plots show platelet counts with median ± IQR, group separation was performed according to median platelet count (300,000/µL). **(B)** Overall survival (KRT/death) within 40 days after admission according to group separation by median platelet count (300,000/µL). Comparison of survival curves was performed with log rank (Mantel-Cox) testing. **(C)** Probability of discharge from hospital after admission according to group separation by median platelet count (300,000/µL). Comparison of survival curves was performed with log rank (Mantel-Cox) testing. ANCA, antineutrophil cytoplasmic antibody; CI, confidence interval; HR, hazard ratio; IQR, interquartile range; KRT, kidney replacement therapy.

Finally, we performed cohort dichotomization by using median platelet counts (300,000/µL) to separate groups for survival-curve analyses using the Kaplan-Meier method and log-rank testing (**Figure 3A**). As previously observed, group separation resulted in significant differences were again observed for markers of kidney injury, including serum creatinine levels (*p=0.0007*), eGFR (*p=0.0029*), and BUN (*p=0.0061*), and complement C3 levels (*p=0.0313*, **Table 2**). This also correlated with disease course reflected by significant differences in requirement of kidney replacement therapy (KRT) or death in the subgroup below median platelet counts (HR: 4.1, 95% CI: 1.6-10, *p=0.0047*, **Figure 3B**). Furthermore, we observed a lower probability of discharge with prolonged hospitalization in this subgroup (HR: 0.5, 95% CI: 0.3-0.9, *p=0.0113*, **Figure 3C**), indicating disease course and recovery in ANCA-associated renal vasculitis differed according to platelet counts.

**Table 2:** Group separation by median platelet count (300,000/µL).

Clinical data	<median	*≥*median	p value
Age – years Female sex – no. (%) MPO-/PR3-ANCA – no./no. (%/%) BVAS – points	65 (54.5-76) 9 (34.6) 16/10 (61.5/38.5) 17 ± 4.4	65 (54-74) 14 (51.9) 10/17 (37/63) 18.6 ± 3.9	*0.4930* *0.2056* *0.0745* *0.1725*
*Serum parameters*			
Platelets – x1,000/µL INR –ratio aPTT – seconds Fibrinogen – mg/dL Hemoglobin – g/dL WBC – x1,000/µL Creatinine – mg/dL eGFR – mL/min/1.73 m^2^ BUN – mg/dL Potassium – mmol/L CRP – mg/L Albumin – g/dL Complement C3 – g/L Complement C4 – g/L ALT – U/L AST – U/L γGT – U/L AP – U/L Bilirubin – mg/dL Lipase – U/L LDH – U/L	207 (159.8-258.8) 1 (1-1.2) 28 (26-31.5) 388 ± 155.4 9.3 (8.15-10.8) 9.88 (6.74-14.1) 3.62 (2.11-5.01) 16.25 (9.03-27.4) 53 (36.5-77.5) 4.3 (4.15-4.7) 60.4 (6.55-113.2) 2.08 ± 0.6 1.12 ± 0.3 0.25 ± 0.1 13.5 (10-25.5) 25 (15-28.5) 54 (17-77.75) 86.5 (71-106.5) 0.4 (0.3-0.55) 46 (25.5-70.5) 265.5 (210.3-301.5)	414 (347-587) 1.1 (1-1.3) 28 (26.75-32) 390.1 ± 232.7 10.2 (9.2-12.2) 12.4 (9.86-15.5) 1.42 (0.77-2.91) 48.4 (24-98.4) 29 (19.5-44) 4.3 (4-4.43) 63.8 (26.2-109.5) 2.76 ± 0.5 1.35 ± 0.28 0.25 ± 0.08 19 (8-47) 21 (18.75-34) 34 (20-107) 90 (70-126.5) 0.3 (0.3-0.73) 24 (19.5-30.5) 288.5 (241.5-326.8)	*<0.0001* *0.5208* *0.7177* *0.9805* *0.1106* *0.0672* *0.0007* *0.0029* *0.0061* *0.3401* *0.4037* *0.0110* *0.0313* *0.8862* *0.8525* *0.9856* *0.9955* *0.4653* *0.8660* *0.0563* *0.2901*

Mean ± SD are shown for normally distributed values, group comparisons were performed with unpaired student’s t-test. Median (IQR) for non-normally distributed values, group comparisons were performed with Mann-Whitney-U-test. Categorical variables are presented as frequency and percentage, non-parametric between-group-comparisons were performed with Pearson’s Chi-square test.

ALT, alanine aminotransferase; ANCA, antineutrophil cytoplasmic antibody; AP, alkaline phosphatase; aPTT, activated partial thromboplastin time; AST, aspartate amino transferase; BUN, blood urea nitrogen; BVAS, Birmingham Vasculitis Activity Score; CRP, C-reactive protein; eGFR, estimated glomerular filtration rate (CKD-EPI); INR, international normalized ratio; IQR, interquartile range; LDH, lactate dehydrogenase; MPO, myeloperoxidase; no., number; PR3, proteinase 3; SD, standard deviation; WBC, white blood cells; γGT, gamma glutamyl transferase.

## Discussion

Innate immunity refers to non-specific recognition of exogenous or endogenous components, leading to an activation of the immune system, and resulting in clearance of these molecules. Megakaryocyte-derived platelets are a key component of the blood, playing a critical role in immune responses by secretion of active mediators. The ability of enucleated platelets to crosstalk with immune cells contributing to innate immune responses is attributed to the expression of membrane proteins, storage granules, and a variety of megakaryocyte-derived molecules. Platelets are a key component of innate immunity that trap immunogenic molecules by necessitating the process of clotting, preventing them from being accessible and to disseminate. Moreover, it is also well established that the platelets are the primary mediators of hemostasis and thrombosis. Therefore, it is increasingly recognized that a hypercoagulable state is persistently active in patients with ANCA-associated renal vasculitis, and studies investigating the impact of tailored anticoagulation are needed to reduce the burden of thromboembolism (24).

We here observed that most patients with active ANCA-associated renal vasculitis had platelet counts within or above the normal range. This is in line with previous reports that platelet counts were significantly higher in patients in active disease stage compared with those in remission (44). Within our cohort of active ANCA-associated renal vasculitis, we observed an association between lower platelet counts and kidney injury, disease course and recovery in ANCA-associated renal vasculitis. These results implicate that platelets in active ANCA-associated renal vasculitis result from a reactive increase, and that a consumptive decrease may indicate accelerated kidney injury. Interestingly, this concept is confirmatory to previous reports that hypercoagulability contributes to kidney injury in vascular diseases, including ANCA-associated renal vasculitis (45–48). In addition, we here observed that lower platelet counts associated specifically with tubulointerstitial injury in ANCA-associated renal vasculitis independent of any glomerular lesion. In this context, experimental studies have already shown that platelets are main contributors to microvascular injury and rarefaction of tubulointerstitial capillaries (49). In ANCA-associated renal vasculitis, the occurrence of tubulointerstitial injury independent of glomerular lesions has already been described (50–53). Our observations might indicate that the tubulointerstitial compartment is particularly susceptible to a hypercoagulable state mediated by platelets. The concept that loss of kidney function is more affected by tubulointerstitial rather than glomerular injury has been described more than 50 years ago in glomerulonephritis (54). In response to tubular damage, different cell death modes (namely apoptosis, necroptosis, and ferroptosis) feature different degrees of immunogenicity, wherein the two latter-mentioned constitute different pathways that are implied in acute kidney injury (AKI), transition from AKI to CKD, sepsis-associated AKI, but also autoimmune vasculitis affecting the kidneys (55).

Moreover, lower platelet counts associated with complement system activation reflected by lower serum C3 levels in ANCA-associated renal vasculitis. Complement system activation with reduced complement C3 levels and intrarenal complement deposits are important denominators of poor outcome in ANCA-associated renal vasculitis (56–60). Our observation that an association between platelets, kidney injury and complement C3 is not present in disease controls (IgA nephropathy, diabetic kidney disease, and acute interstitial nephritis) and specifically observed in the MPO-ANCA subgroup implicates distinct pathomechanisms dependent on seropositivity for MPO-ANCA or PR3-ANCA. This is especially relevant since two complement system inhibitors are currently in clinical development for AAV. Thus, our results may contribute to a more personalized treatment approach of AAV depending on the ANCA serotype and the relevance of complement system activation.

Finally, we here provide evidence that platelets and complement system activation is especially relevant in the subgroup with MPO-ANCA seropositivity. Detailed morphological analysis of tubular injury indicated that lower platelet counts correlated specifically with tubular dilatation in renal vasculitis with MPO-ANCA seropositivity, while an association with cellular casts was observed in the PR3-ANCA subgroup. Although tubular dilatation and cellular casts are both observed in injured kidneys indicating tubular injury and cell death, the association with either ANCA subtype again could also implicate distinct pathomechanisms of tubulointerstitial injury dependent on seropositivity for MPO-ANCA or PR3-ANCA (61, 62). Regarding potential mechanisms, it has been suggested that MPO can directly interact and induce priming of platelets that can contribute to the development of vascular inflammation (63). Therefore, binding of MPO-ANCA autoantibodies could further aggravate platelet activation and vascular injury. These results require confirmation but may contribute to a personalized treatment approach of AAV.

Our study has several limitations, such as the small patient number and the retrospective study design. Furthermore, our observations are associative and do not proof causality requiring mechanistic studies that also include platelet activation and function. Nevertheless, we provide a clinically approached link between platelets, complement system activation and tubulointerstitial injury patterns specifically in MPO-ANCA-associated renal vasculitis, thus broadening our current pathophysiological understanding.

## Conclusion

Based on our observation that an association between platelets and complement system activation is only observed in the MPO-ANCA subgroup, this could implicate that platelets and complement C3 link innate immunity to tubulointerstitial injury in the presence of MPO-ANCA autoantibodies. These results require confirmation but may contribute to a personalized treatment approach of AAV.

## Data availability statement

The original contributions presented in the study are included in the article/**Supplementary Material**. Further inquiries can be directed to the corresponding author.

## Ethics statement

The studies involving human participants were reviewed and approved by the local Ethics committee (no. 22/2/14 and 28/09/17). The patients/participants provided their written informed consent to participate in this study.

## Author contributions

BT conceived the study and edited the manuscript. EB collected and analyzed data, and wrote the first draft. DT collected and analyzed data, IK and SH evaluated kidney biopsies. All authors contributed to the article and approved the submitted version.

## Funding

This study was supported by the Else-Kröner research program entitled “*molecular therapy and prediction of gastrointestinal malignancies*”, grant number 7-67-1840876. We also acknowledge support by the Open Access Publication Funds of the Göttingen University. The funders had no role in the design of the study; in the collection, analyses, or interpretation of data; in the writing of the manuscript, or in the decision to publish the results.

## Conflict of interest

Author SH was employed by company SYNLAB Pathology Hannover, SYNLAB Holding Germany.

The remaining authors declare that the research was conducted in the absence of any commercial or financial relationships that could be construed as a potential conflict of interest.

## Publisher’s note

All claims expressed in this article are solely those of the authors and do not necessarily represent those of their affiliated organizations, or those of the publisher, the editors and the reviewers. Any product that may be evaluated in this article, or claim that may be made by its manufacturer, is not guaranteed or endorsed by the publisher.
