# Supplementary material for: Implication of platelets and complement C3 as link between innate immunity and tubulointerstitial injury in renal vasculitis with MPO-ANCA seropositivity
Source: Front Immunol. 2022 Nov 11;13:1054457. doi: 10.3389/fimmu.2022.1054457 (PMC9692128; doi:10.3389/fimmu.2022.1054457)
Supplement: Supplementary file 1 [file DataSheet_1.pdf]

Implication of platelets and complement C3 as link between innate immunity and tubulointerstitial injury in renal vasculitis with MPO-ANCA seropositivity

Supplementary Material

Eva Baier[1], Désirée Tampe[1], Ingmar Alexander Kluge[2], Samy Hakroush[2,3], Björn Tampe[1,#]

[1]*Department of Nephrology and Rheumatology, University Medical Center Göttingen, Göttingen, Germany*

[2]*Institute of Pathology, University Medical Center Göttingen, Göttingen, Germany*

[3]*SYNLAB Pathology Hannover, SYNLAB Holding Germany, Augsburg, Germany*

[#]*Corresponding author*

Running title: Platelets and C3 in ANCA-associated renal vasculitis

Corresponding author

Björn Tampe, MD

Department of Nephrology and Rheumatology

University Medical Center Göttingen

Georg August University

Göttingen, Germany

Email: bjoern.tampe@med.uni-goettingen.de

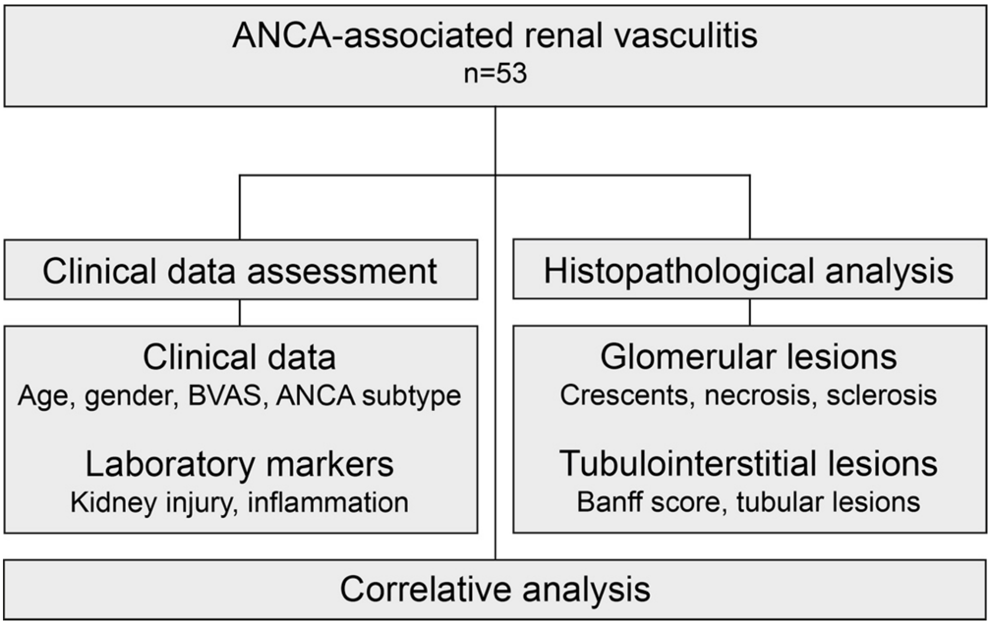

Supplementary Figure 1. STROBE flow chart of study conduction.

Abbreviations: Abbreviations: ANCA, antineutrophil cytoplasmic antibody; BVAS, Birmingham Vasculitis Activity Score; STROBE, Strengthening the Reporting of Observational Studies in Epidemiology.

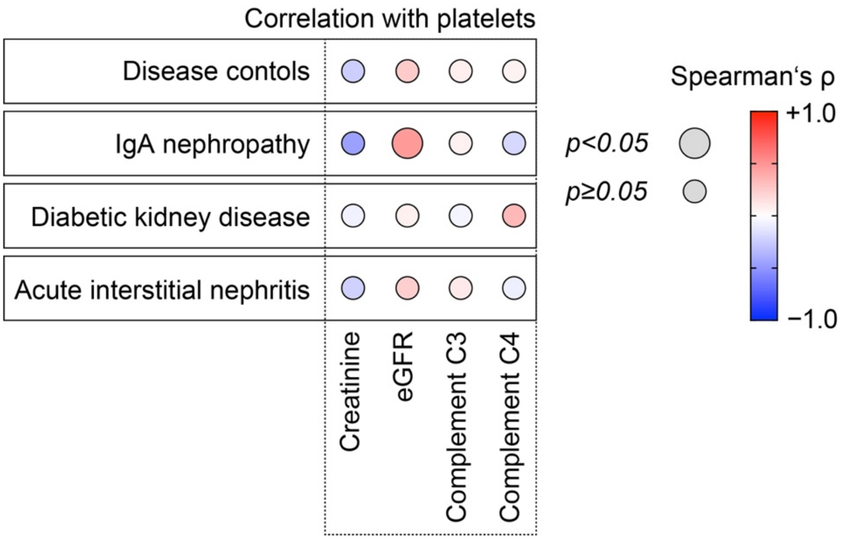

Figure 2. No association between platelet counts, kidney injury and complement in disease controls.

Correlations between platelet counts, serum creatinine, eGFR, complement C3 and C4 in disease controls including IgA nephropathy, diabetic kidney disease and acute interstitial nephritis are shown by heatmap reflecting mean values of Spearman's ρ, circle size represents significance level.

Abbreviations: eGFR, estimated glomerular filtration rate (CKD-EPI).

Supplementary Table 1. Characteristics of the total cohort of ANCA-associated renal vasculitis.

Clinical data	*Value*
Age – years	65 (54.5-74.5)
Female sex – no. (%)	23 (43.4)
MPO-/PR3-ANCA – no./no. (%/%)	26/27 (49.1/50.9)
BVAS – points	17.9±4.2
Serum parameters	
Platelets – x1,000/µL	300 (207-438)
INR –ratio	1 (1-1.2)
aPTT – seconds	28 (26-32)
Fibrinogen – mg/dL	389±193.1
Hemoglobin – g/dL	9.6 (8.65-11.4)
WBC – x1,000/µL	11.2 (8.35-14.85)
Creatinine – mg/dL	2.16 (1.08-4.29)
eGFR – mL/min/1.73 m^2	24.6 (12.65-61.7)
BUN – mg/dL	43.5 (25-66.75)
Potassium – mmol/L	4.3 (4.1-4.5)
CRP – mg/L	63.4 (15.6-110.2)
Albumin – g/dL	2.32±0.65
Complement C3 – g/L	1.24±0.31
Complement C4 – g/L	0.25±0.09
ALT – U/L	17 (9.5-45)
AST – U/L	23 (18-31)
γGT – U/L	46 (20-88)
AP – U/L	87 (71-117)
Bilirubin – mg/dL	0.4 (0.3-0.7)
Lipase – U/L	29 (21.5-48.5)
LDH – U/L	280 (236-324.3)
Initial treatment	
Use of PEX – no. (%)	20 (37.7)
Sessions of PEX – no.	10 (18.9)
Intravenous steroid pulse – no. (%)	37 (69.8)
Oral steroids – no. (%)	53 (100)
Further remission induction	
RTX – no. (%)	19 (35.8)
CYC– no. (%)	25 (47.2)
RTX/CYC – no. (%)	8 (15.1)
Other – no. (%)	1 (1.9)

Mean±SD are shown for normally distributed values, median (IQR) for non-normally distributed values.

Abbreviations: ALT, alanine aminotransferase; ANCA, antineutrophil cytoplasmic antibody; AP, alkaline phosphatase; aPTT, activated partial thromboplastin time; AST, aspartate amino transferase; BUN, blood urea nitrogen; BVAS, Birmingham Vasculitis Activity Score; CRP, C-reactive protein; CYC, cyclophosphamide; eGFR, estimated glomerular filtration rate (CKD-EPI); INR, international normalized ratio; IQR, interquartile range; LDH, lactate dehydrogenase; MPO, myeloperoxidase; no., number; PEX, plasma exchange; PR3, proteinase 3; RTX, rituximab; SD, standard deviation; WBC, white blood cells; γGT, gamma glutamyl transferase.

Supplementary Table 2. Characteristics of disease controls.

IgA nephropathy	*Value*
Platelets – x1,000/μL	247±74
Age – years	57.7±15.6
Female sex – no. (%)	8 (44.4)
Creatinine – mg/dL	1.52 (0.96-4.14)
eGFR – mL/min/1.73 m^2	48.7±33.8
Complement C3 – g/L	1.15±0.25
Complement C4 – g/L	0.27±0.08
Diabetic kidney disease	
Platelets – x1,000/μL	266±82
Age – years	65.5 (53.8-72)
Female sex – no. (%)	7 (35)
Creatinine – mg/dL	2.82±1.53
eGFR – mL/min/1.73 m^2	25.5 (13.5-39.8)
Complement C3 – g/L	1.25±0.31
Complement C4 – g/L	0.34±0.09
Acute interstitial nephritis	
Platelets – x1,000/μL	246±90
Age – years	70 (58-75)
Female sex – no. (%)	8 (29.6)
Creatinine – mg/dL	2.8 (2.23-6.84)
eGFR – mL/min/1.73 m^2	22 (8-31)
Complement C3 – g/L	1.14±0.23
Complement C4 – g/L	0.28±0.1

Mean±SD are shown for normally distributed values, median (IQR) for non-normally distributed values.
Abbreviations: eGFR, estimated glomerular filtration rate (CKD-EPI); IQR, interquartile range; no., number; SD, standard deviation.
